# Evaluation of Digestion Methods in Microplastic Recovery from Mussels (*Mytilus galloprovincialis*) for a Standardised Microplastic Isolation Protocol

**DOI:** 10.3390/foods14223853

**Published:** 2025-11-11

**Authors:** Flavia Capuozzo, Nicoletta Cristiana Quaglia, Angela Di Pinto, Michele De Rosa, Federica Ioanna, Angela Dambrosio

**Affiliations:** 1 Unit of Safety of Food of Animal Origin, Department of Veterinary Medicine, University of Bari Aldo Moro, 70010 Valenzano, Italy; flavia.capuozzo@uniba.it (F.C.); angela.dipinto@uniba.it (A.D.P.); m.derosa1986@gmail.com (M.D.R.); angela.dambrosio@uniba.it (A.D.); 2 Competent Authority for Food from Animal Origin Inspection, Azienda Sanitaria Locale Bari, 70124 Bari, Italy; fedeioanna@hotmail.com

**Keywords:** microplastics, bivalve molluscs, digestion methods, food safety, risk assessment

## Abstract

Although microplastics are known as bivalve mollusc contaminants, the standardisation of isolation protocols hasn’t been developed yet. This study aims at assessing the best microplastic recovery rates and digestion efficiencies, applying two chemical reagents (10% KOH and 30% H_2_O_2_) across a wide range of known temperatures, on mussels (*Mytilus galloprovincialis*) contaminated with virgin microplastic standards. Both reagents provided good digestion efficiencies, but microplastic recovery was optimised employing 30% H_2_O_2_ at 50–60 °C, which also preserved polymer integrity. Indeed, recovery rates ranged from 88.75 to 91.86% at 50 and 60 °C, respectively, whereas 85.8 and 99.4% were the values of the digestion efficiency at 50 and 60 °C, respectively. Flotation and supernatant fractionation were found to be decisive parameters in maximising microplastic recovery; therefore, they shouldn’t be overlooked. These findings lay the foundations for standardising microplastic isolation protocol from mussels, allowing for the reproducibility of data and consequently the comparison of different laboratories’ results in order to concretely assess the risk for consumer health and lead to determining the benchmarks for food safety policymaking. Further studies are needed to standardise the method for the detection of microplastics from other foods.

## 1. Introduction

Microplastics (MPs) have been documented to be a contaminant occurring in a wide range of foods. Hence, potential health risks that may result from regular human consumption might be inevitable. Understanding the impacts of the presence of microplastics in food is essential for human health risk assessment and a key solution to addressing this global issue [1]. Nevertheless, to date a standard protocol to detect these microplastic particles hasn’t been developed yet, due to the numerous variables linked to the nature of the food matrix, the large variety of chemicals that can be employed to digest the organic matter, the optimal temperature for the digestion, exposure times, the possibility to include a density separation phase (i.e., flotation), choosing different densities and different types of salts, quality controls (e.g., blanks, laboratory cleaning, air filtration, cotton lab coats) and various methods for the chemical identification. All these parameters bring both advantages and disadvantages to the recovery of MPs [2].

Bivalve molluscs are the most investigated since they accumulate microplastics filtering large amounts of seawater to feed. Furthermore, they deserve a peculiar focus since they are eaten whole, including all the contaminants such as MPs, thus representing a potential health risk to the consumer [3,4].

To date, most of the MP experimental isolation protocols for bivalve molluscs in the literature include a temperature-assisted digestive phase to disaggregate the biological tissue of samples and a density separation or flotation step where MPs are supposed to be collected in the supernatant that is subsequently filtered. Filtration is followed by filter visual sorting under a stereomicroscope in order to detect and classify the potential plastic items into different categories of shape, colours and dimensions. Finally, chemical identification is used to confirm the results of visual sorting and to determine the type of polymers isolated.

Many authors focused on specific variables in their research on MP occurrence in bivalves. The most investigated is the chemical digestion of the biotic matrix, since it breaks down tissues that can interfere with the visual sorting and the chemical identification of plastic particles [2]. The digestion phase can be conducted by employing proteases, lipases, Corolase 7089, trypsin, proteinase K or a blend of different enzymes [5,6,7,8,9,10,11]. Enzymatic methods are documented to be gentler but also arduous, expensive and time-consuming [12].

Other authors have considered acidic digestion using HNO_3_, HClO_4_ or a combination of different acids, yet assessing the loss of particles due to the overly aggressive action of acids [13,14,15,16,17,18].

Alkaline digestion with 10% KOH or NaOH and wet oxidative digestion with 30% H_2_O_2_ are the most commonly used methods to disaggregate the organic matter [3,19,20,21,22,23,24,25,26,27,28,29]. Nevertheless, using KOH 10% may lead to discolouring certain polymers at high temperatures [30]. Whereas oxidative methods, at high concentrations (H_2_O_2_ ≥ 30%) and long incubation times, can produce excessive foam, reducing digestion efficiency and discolouring plastic particles [29,30]. Additionally, all these methods may also be combined [15,31,32]. Chemical digestion is the most critical phase to recover MPs because it should remove biological matrix components without damaging polymers. This makes it directly related to the recovery of plastic particles. Generally, mechanical agitation (typically 80 rpm) is associated with chemical digestion to enhance the efficiency of complex biological matrix digestion. The digestion phase is influenced by an equally important parameter, namely the incubation temperature. The latter influences both the digestion rate and the recovery rate. Despite its potential impact, temperature is frequently neglected and insufficiently studied within microplastic isolation methods. A common approach in the literature involves single-temperature treatments ranging from 40 °C to 80–100 °C [20,26,33,34,35,36]. Weak thermal conditions (e.g., 40 °C) typically result in an underestimation of plastic particles due to incomplete digestion of organic matter [35]. Similarly, higher temperatures (over 60 °C) also lead to underestimating MPs due to the aggregation or loss of certain polymers [20,26,35,36].

Density separation is often used after chemical digestion to collect MPs in the supernatant and to allow the sedimentation of inorganic particles (like sand) on the bottom. Most authors use sodium chloride (NaCl) or sodium iodide (NaI) solutions at different concentrations [37,38,39]. Salt concentration is crucial to allow the flotation of particles in the supernatant; indeed, choosing lower concentrations may preclude the recovery of denser polymers. NaCl is the most commonly used type of salt, compared to NaI, which is more expensive and may represent a health hazard [40].

Few studies have evaluated the application of Red Nile colourant as a straighter way to detect MPs through visual sorting [8,41,42]. Anyway, the application of Red Nile alone may be limiting because of the influence of variables such as solvent choice, image capturing and analysis, false positives from natural organic matter and limited detectability of highly crystalline and smaller MPs [41].

Finally, chemical characterisation is performed after visual sorting, employing sample-nondestructive methods such as Raman spectroscopy [37,40,41,42], FT-IR [3,20,24,25,26,43,44], and Scanning Electron Microscopy and Energy Dispersive X-ray Spectroscopy (SEM/EDS) [45,46,47,48]; alternatively, sample-destructive methods can be applied, like Pyrolysis-Gas Chromatography-Mass Spectrometry [22,49,50].

This study aimed at recovering the higher number of MPs from bivalve mollusc tissues and assessing the digestion efficiency of the treatments employing oxidative and alkaline digestions across a known range of temperatures. Furthermore, filtrate fractions were evaluated after flotation in order to assess the number of recovered particles for each fraction in relation to polymer densities and morphologies. Mussels (*Mytilus galloprovincialis*) were experimentally contaminated with a known number of virgin MPs. The efficiency of two chemical reagents was evaluated at different incubation temperatures based on the recovery of MP standards. The choice of these methods is motivated by the belief that digestion protocols cannot be evaluated only on the basis of tissue removal. Instead, their reliability must take into account recovery efficiency, polymer preservation, and compatibility with subsequent spectroscopic identification [2]. Applying different temperatures and monitoring both digestion efficiency and recovery, this study provides an integrated assessment of how experimental conditions affect the accuracy of microplastic quantification in complex food matrices such as mussel tissues. Guaranteeing high recovery rates is fundamental to accurately evaluating the potential risks to human health related to the consumption of MP-contaminated bivalve molluscs.

## 2. Materials and Methods

### 2.1. Experimental Design

Six different temperatures, i.e., room temperature, 30, 40, 50, 60 and 70 °C, were investigated, whereas 30% H_2_O_2_ and 10% KOH were selected as the most used reagents for shellfish tissues and employed independently with a subsequent density separation [3,21,35]. Samples were contaminated with virgin MP standards of different sizes, divided into 3 replicates, and incubated at each selected temperature for 24 h with an additional 24 h at room temperature.

### 2.2. Reference Microplastics Selection and Chemical Resistance of Plastics

Secondary microplastics were generated from purple polyethylene (PE) pellets (density 0.91 to 0.94 g/cm^3^) and blue polyamide (PA) fibres (density 1.12 to 1.44 g/cm^3^). Purple and blue colours were selected because of their self-evident detectability during visual sorting after the filtration. Pellets and fibres were cut in the laboratory with a metallic electric grinder and subsequently sieved through stainless steel meshes (AS 200 basic) with known porosity diameters (Retsch, Verder Scientific, Dusseldorf, Germany). MPs ranging from 250 µm to 5 mm were retained and used as experimental standards.

A week before the experiment, a chemical resistance test was conducted by exposing twenty virgin MPs for each standard to 30% H_2_O_2_ and 10% KOH independently at 70 °C for seven days in order to assess their stability under the extreme investigated condition. Resistance was assessed by detecting changes induced by the treatment through visual inspection under a stereomicroscope (Nikon, Moncalieri, (TO), Italy). Potential plastic items were discriminated from non-plastic debris and residual organic matter by employing a fine-tipped needle that assessed the items’ breakability. MPs’ colour, shape, and size before and after exposure were compared. Colour changes were reported applying the Pantone Color Matching System as a standardised palette [51], and images were acquired with a digital camera (X Entry 23.6.1) (Nikon, Moncalieri, (TO), Italy).

### 2.3. Sampling and Sample Preparation

Mussels (*M. galloprovincialis*) were selected as the bivalve mollusc model species. Six samples were purchased at different local retail fish markets in Bari (Apulia region, Italy) in May 2025. Each sample consisted of 60 live mussels, refrigerated and labelled in 1 kg net bags. The scientific species names were reported on the labels. Immediately after transportation, samples were divided into three replicates of 20 individuals, and the analyses were performed independently on each replicate.

### 2.4. Digestion Treatment Conditions and Density Separation

Each mussel was rinsed 3 times with pre-filtered distilled water to remove sediment and debris. They were deshelled, and the soft tissue was pooled to obtain aliquots. From each pool, 10 g was transferred into a 1 L glass bottle, to which 200 mL of 30% H_2_O_2_ or 10% KOH solutions (1:20 *w*/*v*) (Fisher Scientific, Honeywell Fluka, Sagrate, Milano, Italy) was added for organic matter digestion. 40 PE fragments and 40 PA fibres were added to each replicate (80 MPs in total). The flasks were covered with aluminium foil, and each sample was transferred independently into oscillation incubators (80 rpm) set with the temperatures under investigation.

Incubation at the designed temperatures (room temperature—RT, 30, 40, 50, 60, and 70 °C) lasted 24 h, followed by an additional 24 h at room temperature. In the first 24 h, samples were monitored to prevent potential heat rise due to the chemical reactivity at high temperatures [52].

Two positive blank samples were prepared by adding the same number of MPs as the samples to the digestion reagents without biological tissue to evaluate the effect of the chemical solutions alone in order to exclude the interference of the organic matrix on the particle recovery.

Density separation was performed by preparing a hypersaturated solution of NaCl (1.2 g mL^−1^) dissolving the salt in distilled water, and mechanically agitating it for 24 h at room temperature. The saline solution was filtered, and ≈800 mL was added per sample in 1 L cylinders containing the digestates; then they were rapidly covered with aluminium foil and maintained at room temperature overnight.

### 2.5. Filtration, Digestion Efficiency and Recovery Rate

For each sample, particle distribution was observed across all digestate layers in the flotation cylinder; thereafter, the supernatant (≈150 mL) was filtered through membrane filters (pore size: 1.2 μm; diameter: 47 mm) (Axiva Sichem Biotech, Delhi, India) using a Membrane–Laborpunpe (KNF Flodos AG, Sursee, Switzerland) under vacuum. Every 50 mL, the filter was replaced with a new one, obtaining sequential 50 mL aliquots of filtrate (i.e., 50 mL, 100 mL, 150 mL). All the filters collected were placed in pre-rinsed glass Petri dishes, covered, and dried at room temperature. Nitrocellulose and glass fibre filters were employed for H_2_O_2_ and KOH, respectively.

Purple PE fragments and blue PA fibres were detected and observed under a stereomicroscope. Particle colours before and after filtration were compared, and images were acquired with a digital camera.

*Digestion efficiency* (%) was calculated as:

(1) $$Digestion\;efficiency\;\left(\%\right)=\frac{\left[Wi-\left(Wa-Wb\right)\right]}{Wi}\times 100$$

where *Wi* = Initial weight of biological materials (g), *Wa* = Weight of dry filter membrane after filtration (g), and *Wb* = Weight of dry filter membrane before filtration (g) [30].

MP *Recovery rate* (%) was calculated as the number of extracted particles divided by the number of added particles:

(2) $$\;Recovery\;rate\;\left(\%\right)=\frac{Nf}{Ni}\times 100$$

where *Nf* = Number of microplastics extracted in each sample after filtration, and *Ni* = Number of microplastics added to each sample [34].

### 2.6. Quality Assurance/Quality Control Measures

To minimise contamination, strict precautions were implemented during the entire experiment.

All staff members wore a white 100% cotton lab coat and nitrile gloves all the time; the laboratory door remained closed, and movements in and out of the room were kept as minimal as possible. All laboratory equipment was made of glass or metal and rinsed three times with filtered distilled water before and after use and covered with aluminium foil to prevent airborne MP contamination. Work surfaces were kept clean using 100% cotton towels soaked in 90% filtered ethanol.

All fluids employed during the experiment (distilled water, saline solution, potassium hydroxide and hydrogen peroxide) were filtered before use with membrane filters with a pore size of 0.45 μm and a diameter of 47 mm (Axiva Sichem Biotech, Delhi, India) and kept in pre-rinsed glass flasks covered with aluminium foil.

### 2.7. Statistical Analysis

Data were analysed using Microsoft Excel with a significance level of α = 0.05. Results are reported as mean values of replicates with standard deviation (SD).

Mean values of replicates and standard deviations were calculated for each sample. Both the digestion efficiencies of mussel tissues and the recovery rates of MPs were calculated in order to evaluate the efficiency of the digestion at different temperature conditions. Non-parametric Friedman and Quade tests were used to assess the overall recovery rates of each treatment.

Post hoc comparisons were performed applying Dunn’s test with Bonferroni correction and the Nemenyi test, which compared treatment performance across 50, 100, and 150 mL filtered supernatant volumes and the number of recovered fragments and fibres. The Wilcoxon signed-rank test assessed whether chemical reagents selectively affected morphology recovery at each temperature. The Kruskal–Wallis test compared microplastic dimensions between chemical solutions; finally, the Mann–Whitney U test was performed to assess the potential influence of supernatant volume fractions on MP dimensions.

## 3. Results

The chemical resistance test results in no visible change in shape, size and colour after independent exposure of MP standards to 30% H_2_O_2_ and 10% KOH alone for seven days. Digestion efficiency was calculated for both reagents and showed an increase in values closely correlated with the increase in temperature. At 70 °C, almost complete tissue digestion was achieved for both chemical reagents. For the 30% H_2_O_2_ solution, the results showed a digestion efficiency of 59.8% at room temperature and an increase in the digestion rate of approximately 10 percentage points as the temperature increased, up to a maximum of 99.3% at 70 °C. 10% KOH showed slightly higher digestion efficiency rates, with values of 63.6% at room temperature and of 99.4% already at 60 °C.

**Figure 1:**
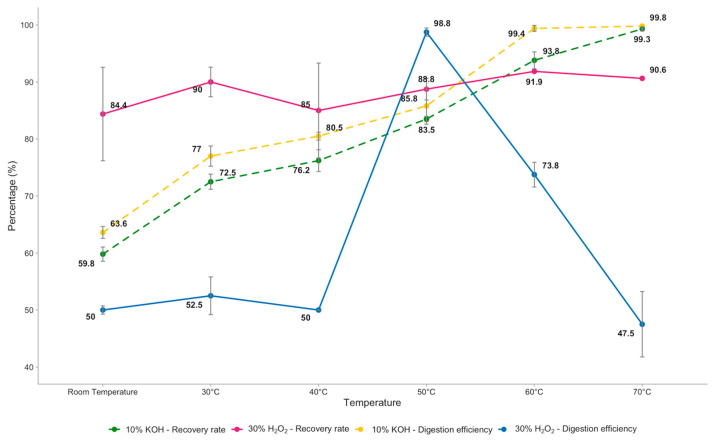
Digestion efficiencies and recovery rates of 30% H_2_O_2_ and 10% KOH at all temperatures applied.

Total recovery rates were calculated for both reagents (Figure 1).

H_2_O_2_ showed a stable and gradual increase across temperatures, with recoveries ranging from 84.38% to 90.63%. In contrast, 10% KOH exhibited highly inconsistent performance; recovery remained low from room temperature to 40 °C (≈50%), peaked at 50 °C (98.75%), and then sharply declined at higher temperatures, dropping below 50% at 70 °C. Although recovery rates differed, the Friedman test showed no statistically significant differences in the number of recovered particles between the two reagents at the same temperature. The Quade test showed that 30% H_2_O_2_ had a higher influence on the number of microplastics recovered across all temperatures (10% KOH W = 6; 30% H_2_O_2_ W = 10.5), with mean values of MPs recovered between 68 ± 6.56 and 73 ± 7.51 at room temperature and 70 °C, respectively. On the other hand, the maximum number of plastic particles recovered with 10% KOH was 78 ± 0.58 at 50 °C, and the lowest mean value was at 70 °C with 38 ± 4.58 MPs (Table 1).

**Figure 2:**
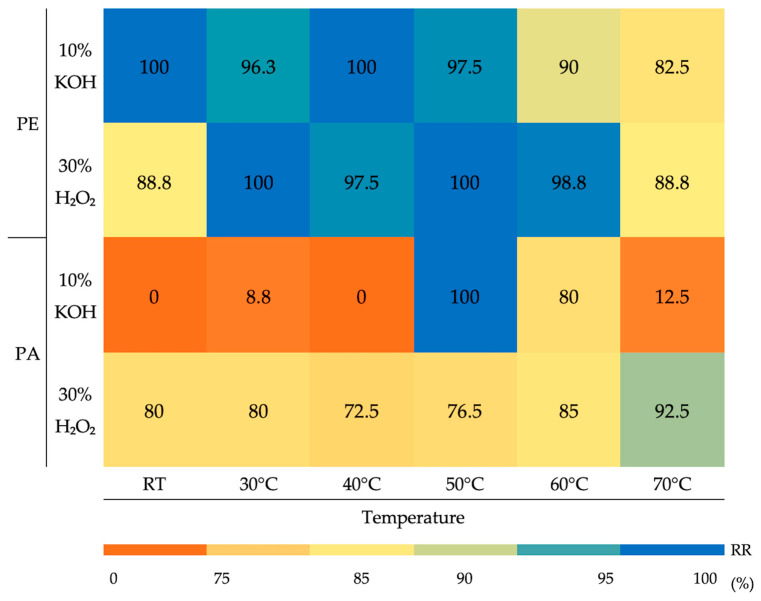
Recovery rates of PE fragments and PA fibres at each treatment applied.

Despite these patterns, the efficiency of microplastic recovery varied significantly depending on the morphotype, digestion reagent, and incubation temperature. Both digestion methods ensured consistently high recovery rates of PE fragments, whereas PA fibres showed a markedly different behaviour (Figure 2).

The application of 10% KOH provided PE fragment recoveries close to 100% from room temperature (RT) up to 50 °C, then slightly declined, reaching 82.5% at 70 °C. Similar stability was observed with 30% H_2_O_2_, where recoveries ranged from 88.8% to 100% across all conditions. Conversely, PA fibre recoveries highly depended on the treatment. 10% KOH determined poor recovery at lower temperatures (from 0 to 8.8% from RT to 40 °C), increased significantly only at 50 °C to 100%, then dropped again to 12.5% at 70 °C. When treated with 30% H_2_O_2_, fibre recovery showed more stability, remaining relatively high at most temperatures, with a peak at 70 °C (92.5%).

**Figure 3:**
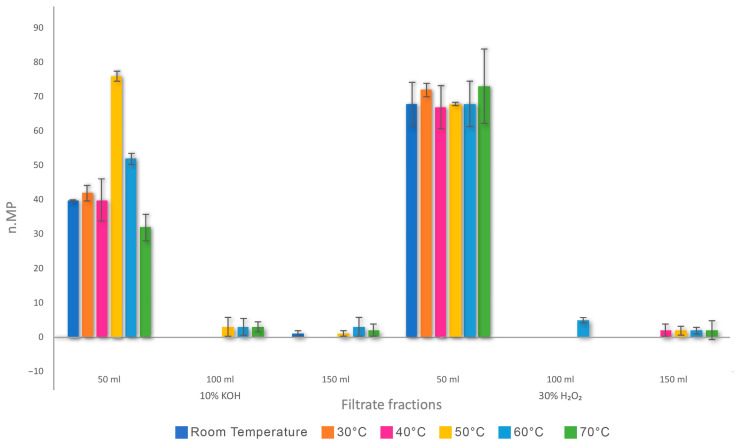
Mean number and SD of recovered MPs at each level of filtration for each treatment applied.

MPs were also quantified in sequential 50 mL aliquots of filtrate (50 mL, 100 mL, 150 mL). Most particle number was recovered in the first 50 mL fraction at all temperatures for both reagents, with subsequent filtrates contributing minimally to total recovery. This difference was statistically significant, as confirmed by Dunn’s test with Bonferroni correction (*p* = 0.02; *p* = 0.003). The Nemenyi post hoc test identified the 50 mL filtrate fraction as the most significant in the number of recovered particles compared to the 100 and 150 mL fractions (CD = 1.35) (Figure 3).

The Wilcoxon signed-rank test showed that PE fragment recovery was consistently high across all digestion protocols, with no statistically significant differences between KOH and H_2_O_2_ at any temperature. In fact, with both reagents, fragments in the 50 mL fraction were consistently higher, whereas the 100 mL and 150 mL fractions contributed marginally to recovery regardless of the temperature applied (Table 2).

Fibre recovery in the 50 mL fraction was high and more stable for H_2_O_2_ than KOH, while it dropped in subsequent filtrates with minimal recovery (Table 2). Across all temperatures, digestion with 30% H_2_O_2_ recovered significantly more fibres than 10% KOH (*p* = 0.009). The only exception was at 50 °C, where KOH achieved complete fibre recovery, while H_2_O_2_ recovered only 31 ± 1.52 fibres.

Although not statistically significant, the Wilcoxon signed-rank test also suggested a trend favouring H_2_O_2_ in MP recovery, confirming its superiority under the tested conditions when compared to KOH.

The different treatments and the filtrate fractions also impacted the recovery of different sizes of MPs (Table 3).

The Kruskal–Wallis test found no significant differences in minimum or maximum MP dimensions between KOH and H_2_O_2_ treatments. Thus, neither the applied reagents nor the different temperatures influenced the dimensions of recovered particles.

Whereas, statistically significant differences were found across the filtrate fractions (Mann–Whitney U, U = 1; critical U = 5). In fact, when 10% KOH was applied from room temperature to 50 °C the largest microplastics were found mainly in the first 50 mL fraction, with smaller particles in subsequent fractions. At 60 °C and 70 °C, the maximum dimensions slightly decreased in the 50 mL fraction but stayed relatively high in the 150 mL fraction. With the application of H_2_O_2_, larger maximum sizes were recovered in the 50 mL fraction with a peak at intermediate temperatures (40 °C) and in the 150 mL fractions.

## 4. Discussion

Given the potential human ingestion of MPs through seafood consumption, bivalve molluscs’ ability to accumulate plastic particles in their tissues has raised concerns about global food safety [24,53,54,55,56]. Therefore, an accurate quantification of MPs requires digestion protocols that efficiently remove biological matrices while keeping undamaged the structure of different polymer types. To maximise recovery and, most importantly, to obtain a reliable evaluation of contamination levels, the choice of chemical reagents, temperature, and procedural parameters is crucial [57].

Since mussels (*M. galloprovincialis*) account for approximately 13% of global marine bivalve production, they were selected as a model species for this study [58]. They are an excellent food for the human diet, as they are a good source of iron, long-chain omega-3 fatty acids, phytosterols, vitamin B12, and high-quality proteins [59]. However, due to their extensive distribution and filter-feeding behaviour, they are also particularly vulnerable to MP contamination, which raises concerns about food safety worldwide [60]. Therefore, it is crucial to develop standardised methods to recover MPs in order to prevent underestimating the number of plastic particles and to provide a solid foundation to assess the risks to human health related to the consumption of MP-contaminated mussels [3,19,24,25].

### 4.1. Polymers Selection

For experimental analysis, mussels (*M. galloprovincialis*) were contaminated with purple PE fragments and blue PA fibres. PE was selected because it is the most widely produced polymer globally, accounting for nearly 50% of all microplastics in the environment due to its strong hydrophobicity (contact angle 85°), chemical stability, and very low biodegradability [61], which make it suitable for various applications, including agriculture, food packaging, and fishing gear [62]. Remarkably, even the net bags containing the mussel samples in this study were made of low-density polyethylene (LDPE), as indicated on their labels, illustrating its ubiquity in seafood packaging.

PA, instead, is a widely produced synthetic polymer, with a global production of approximately 8.7 million tonnes in 2024, mainly dominated by PA6 and PA66 (8.4 million tonnes combined) [63]. Fibres are the predominant form of PA, contributing to microfibre pollution of marine surface waters [64,65,66].

PA’s structural stability offers excellent tensile strength, toughness, and resistance to wear, impact, and extreme temperatures, explaining its widespread use in demanding industrial applications, including the automotive, electrical, and packaging sectors. Increasingly, PA is blended with other polymers to enhance its durability and resistance to extreme temperatures and chemical reagents. For instance, composites of PA12 are used in the biomedical field to create customised implants and prosthetics due to their stability under chemical and physical stress [67].

A week prior to the experiment, in order to assess the resistance of MP standards, a chemical resistance test was performed by exposing our MPs to 30% H_2_O_2_ and 10% KOH separately at 70 °C for seven days.

Results on PE fragments aligned with the Chemical Resistance of Plastic Polymers whose data stated that this polymer is highly resistant to 30% H_2_O_2_ [68]. By contrast, PA is defined as resistant to 10% KOH but not 30% H_2_O_2_. Moreover, Koutny et al. (2006) [69] observed that PE is resistant to oxidation due to its content of antioxidant stabilisers. These substances are present, even in minimal amounts, in all commercial preparations.

Observing the MP standards before and after the resistance test, no changes in colour were remarked with both reagents after the comparison to standardised colour palettes commonly used in the literature [55]. Thus, the influence of the experimental methods on the colour was considered negligible. In addition, no other structural changes were observed after the exposure.

The size range of MP standards was between 250 μm and 5 mm. This is due to the limit of the electric metallic grinder that cut the pellets. Nevertheless, MPs in the 250–500 μm size range are consistent with the dimension category most recovered in many studies [3,19,24,25,66].

### 4.2. Wet Oxidative and Alkaline Treatments for Microplastic Isolation

Our experiment tested 10% KOH and 30% H_2_O_2_ across various temperatures (room temperature, 30, 40, 50, 60, and 70 °C) for 24 h, associated with an additional 24 h at room temperature in order to maintain the action of chemical reagents and mitigate microplastic alteration due to the prolonged combination of high temperature and chemical reagents, thus maintaining recovery accuracy.

Wet oxidative and alkaline treatments are the most commonly used solutions to digest bivalve and fish tissues. 30% H_2_O_2_ acts as a mild oxidative agent, digesting proteins, lipids, and carbohydrates, generally preserving synthetic polymers [34,70]. Some studies [71] have shown that 30% H_2_O_2_ does not cause significant fragmentation of MPs at temperatures below 60 °C and results in only minor changes in the infrared spectral profile. Therefore, it is considered a nondestructive method both chemically and physically, making it efficient for organic matter digestion. This reliability helps reduce analytical bias caused by polymer degradation [57]. Conversely, 10% KOH is known for its strong digestion capacity. It determines saponification and hydrolysis reactions that efficiently degrade tissues but can also alter polymer surfaces, leading to structural weakening or discolouration under certain conditions [72]. Indeed, Dehaut et al. [71] reported KOH to be effective in digesting mussels, although it can cause PA discolouration at 50 and 60 °C with minor impact on chemical identification of the polymer [30]. Despite these limitations, KOH remains widely used because of its efficiency in degrading soft tissues in marine organisms [73,74,75].

Compared to enzymatic or acidic methods, both oxidative and alkaline treatments offer a balance of efficiency and minimal polymer alteration, making them widely adopted in microplastic extraction protocols [30,76].

### 4.3. Recovery Rates, Digestion Efficiencies and Temperature

Digestion efficiencies and MP recovery rates were calculated across a temperature range (room temperature, 30, 40, 50, 60, and 70 °C) for both reagents, according to the formulas proposed by Karami et al. (2017) and Hartmann et al. (2019), respectively [30,51].

The extraction of MPs from mussel tissues demonstrated that polymer type, particle morphology, and reagent selection significantly influence digestion results, with the reagent being an essential factor. De facto, both 10% KOH and 30% H_2_O_2_ improved digestion efficiency with increasing temperature, reaching over 99% tissue removal at 70 °C (Figure 1). Consistent with our findings, the efficiency of alkaline reagents (i.e., KOH) was assessed in *Mytilus edulis* soft tissue, achieving digestion efficiencies ranging from 99.6 to 99.8% at 60 °C; conversely, other researchers preferred the employ of 1 M NaOH as oxidative reagent, resulting in a complete tissue degradation of mussels (100% digestion efficiency), despite the presence of tissue residues at visual inspection of filters [77,78].

The recovery rates showed a remarkable difference. In particular, employing 10% KOH, recovery was low (≈50–52.5%) from room temperature to 40 °C, then sharply increased to 98.8% at 50 °C, decreasing again to 73.8% at 60 °C and 47.5% at 70 °C (Figure 1). Temperatures above 50 °C negatively affected microplastic recovery, likely due to either increased adhesion of microplastics to undigested residual organic matter or partial degradation of specific polymers [51,79].

When exposed to either alkaline or oxidative conditions, PE fragments demonstrated remarkable chemical stability, achieving nearly 100% recovery from room temperature up to 70 °C. A minor decrease was observed at 70 °C with KOH (82%), highlighting PE’s resistance to degradation. This is due to its high crystallinity, low surface area and the presence of antioxidant stabilisers, which preserve stability in oxidative and alkaline environments [69,80]. Anyhow, KOH could almost fully digest tissues but showed variable recoveries, especially above 50 °C, where PA fibre loss increases. This may occur because of fibre aggregation or polymer sinking under alkaline conditions, most likely associated with their larger surface area and tensile loss with consequent higher interactions with digestate [72,81,82,83]. Indeed, during this experiment, fibres were observed settling in lower digestate layers or sediment at the bottom of flotation cylinders. Therefore, KOH-based protocols tend to underestimate microplastic counts. Many studies support this by stating that while protocols relying only on alkaline digestion recover fragments effectively, they often fail to recover fibres, thus underestimating contamination when fibrous particles are predominant and leading to biassed contamination estimates [84,85].

On the other hand, 30% H_2_O_2_ showed consistent recovery rates ranging between 84% and 92% across all temperatures, with minor fluctuations (Table 1). Similar observations have been reported in other studies, where H_2_O_2_ digestion showed high and consistent recovery rates of microplastics from marine organisms [72,86]. Although slightly less efficient than KOH in digesting mussel tissues (Figure 1), its performance appears less sensitive to temperature variations compared to KOH and its stable recoveries make it reliable for isolating microplastics without losing particles, especially under moderate heat (50–60 °C). Its oxidative action degrades proteins and lipids, without affecting the crystalline structure of the polymer, thus leading to more reproducible microplastic quantification [87,88]. Consistent with our results, Li et al. (2023) [89] demonstrated that H_2_O_2_ digestion achieved high recovery rates of textile MP, offering both reproducibility and stable recovery of fibres.

Therefore, moderate heating with KOH can enhance recovery, whereas H_2_O_2_ provides robust and reproducible extraction across a wide temperature range, making it a more reliable reagent for quantitative analyses of microplastics in mussels. Our findings ensured efficient tissue degradation, preventing heat-induced polymer alterations and allowing a good evaluation of digestion protocols based on their suitability to recover microplastic from complex food matrices like bivalve tissues [12]. High digestion efficiency is essential for minimising matrix interference, but the balance with polymer preservation is crucial [90]. Underestimating MPs leads to biassed recovery rates, potentially misleading safety perceptions about the food tested [91]. Obtaining optimal MP recovery requires careful selection of chemical and thermal parameters, especially for delicate particles. Likewise, experimental designs in MP recovery must be selected accurately, since chemical interactions with particles can impact data quality. In fact, the choice of reagents and temperature directly influences the reliability of polymer identification [79]. Standardising these protocols is crucial to ensure comparison between laboratories and to generate accurate data on MP contamination [92].

### 4.4. Influence of Filtration Volume, Temperature, and Particle Size on MP Recovery

Filtration volume proved to be a key factor affecting MP recovery, significantly impacting both particle counts and size distributions.

Employing NaCl for flotation (1.2 g mL^−1^) recovery of low-density MPs improved, especially fragments, by promoting their separation from tissue residues. Floating allows small MPs to remain suspended in supernatant before filtration, reducing particle loss from sedimentation and selective recovery based on buoyancy, thus acting as a pre-filter for low-density particles (like PE, whose density is in a range from 0.91 to 0.94 g/cm^3^) [86,93]. The first filtrate fraction (≈50 mL) captured a more representative portion of MPs compared to subsequent fractions that contained fewer particles (Figure 3). At 50 mL, treatments with KOH and H_2_O_2_ produced high fragment recoveries (34–40 particles) across all temperatures (Table 2), while recoveries decreased sharply at 100–150 mL, often resulting in less than five particles regardless of the reagent used. Fibre recovery showed a similar trend, with the best outputs in the initial 50 mL fraction and remarkable drops in subsequent filtrates. This pattern indicates that particle settling and loss during sequential filtrations can lead to consistent underestimation of MP numbers if sample volumes are not standardised.

Generally, the efficiency of NaCl hypersaturated solution decreases for higher-density polymers like PA, whose densities range from 1.12 to 1.44 g/cm^3^ [93]. For this polymer, the first filtrate fraction may be insufficient, requiring collection and analysis of subsequent filtrates (i.e., 100 mL, 150 mL). Alternatively, higher-density solutions such as sodium iodide or zinc chloride may be needed for complete recovery [94,95]. Nevertheless, our results showed remarkable PA recoveries in the first 50 mL of filtrates when H_2_O_2_ was applied, suggesting that either the density applied to the flotation solution in this experiment was optimal for recovering PA monofilament fibres or the oxidative reagent caused minor alterations of the polymer structure, determining loss of density and consequent buoyancy (as supported by the Chemical Resistance of Plastic Polymers [68]); else, notably, both conditions contributed to PA flotation and the resulting good recovery.

The investigated digestion parameters (i.e., chemical solutions, temperatures and filtration volumes) further influenced particle size recovery and distribution.

Mann–Whitney U tests showed significant effects of filtrate fractions on size distribution, in particular in the pairs 50–100 mL and 50–150 mL for both reagents (U = 1; critical U = 5), while Kruskal–Wallis tests found no significant differences between KOH and H_2_O_2_ in the recovery of minimum or maximum MP sizes (*p* > 0.05). For KOH, minimum sizes were consistent across filtrates, ranging from 0.52 ± 0.98 mm at room temperature to 0.96 ± 0.42 mm at 50 °C (Table 3). Maximum sizes were concentrated in the initial 50 mL fraction and declined at higher digestion temperatures (60–70 °C). These results likely reflect gravity, flow dynamics, polymer aggregation, and partial fibre degradation in alkaline conditions [83]. Strong bases like KOH can cause swelling, discolouration, and fibre degradation in synthetics at high temperatures [57]. H_2_O_2_ digestion showed more stable recovery across fractions and temperatures, with minimum sizes between 0.67 ± 0.11 and 1.27 ± 0.20 mm, and larger maximum sizes than KOH, reaching 5 ± 1.84 mm at 40 °C (Table 3). Dimensions decreased across fractions due to the oxidative mechanism, which degrades tissue but maintains polymer morphology, favouring the detection of smaller MPs [96]. Larger MPs were found mainly in the 50 mL fraction, while smaller particles were consistently found across filtrates.

These findings highlight that parameters like volume fractionation are as important as chemical reagents in determining recovery outcomes. In general, H_2_O_2_ enhanced the recovery of larger fibres and provided a wider size distribution across filtrate fractions compared to KOH. Fragment recovery was less affected by chemical reagents. However, to ensure recovery of polymers of different densities, later filtrate fractions must also be examined.

Overall, our results showed that all parameters, including filtration volumes and flotation, play a critical role in MP recovery and size distribution. A careful selection of reagents and procedural optimisation is fundamental for accurate microplastic analysis, precise exposure assessments and food safety evaluations [97].

### 4.5. Implications for Food Safety

Standardising procedures for the isolation of microplastics from various food matrices is critically needed, as the comparison of digestion applying 10% KOH or 30% H_2_O_2_ showed differences in MP recovery rates due to differences in digestion mechanisms. Differences in recovery rates are also documented by many authors [13,15,18,21,24,29,30,32], indicating that chemical digestion methods highly influence MP number and morphology recovery. H_2_O_2_ efficiently removes organic matter while preserving polymer structure, leading to higher and more consistent recovery. Conversely, KOH may degrade polymers, cause discolouration and reduce tensile strength, resulting in lower and more variable recovery [75,97,98,99,100]. These differences emphasise the importance of reagent choice and suggest that inconsistent protocols contribute to data variability in microplastic studies [84,101,102,103]. Specifically, protocols that maximise tissue digestion (e.g., KOH at high temperatures or acid compounds like HNO_3_ and HClO_4_) may underestimate contamination by damaging fibres, whereas oxidative methods like H_2_O_2_ balance digestion and MP preservation.

MP concentrations may be underestimated if digestion methods and parameters, such as temperature, flotation and supernatant filtration volumes, are not considered or optimised, especially for fibres that are dominant in environmental and food samples [84,101,102]. Underestimating fibre concentrations, often the main microplastic type ingested by humans through seafood, could bias exposure assessments [66,93]. This study shows that H_2_O_2_ digestion at intermediate temperatures (50–60 °C) with selected filtration volumes (e.g., 50 mL) is most reliable for MP quantification in mussels, ensuring full recovery and better contamination assessments [104].

Furthermore, environmental ageing of microplastics must be taken into account when employing an extraction protocol. Indeed, our results recommend mild temperatures (i.e., 50–60 °C) to avoid further degradation of aged microplastics and subsequent underestimation of MP contamination. Since mussels are eaten whole, including the digestive tract, accurately measuring MPs is essential for assessing human dietary exposure and food safety risks [60,76].

Standardising protocols based on reliable conditions, like H_2_O_2_ digestion at 50–60 °C with 50 mL supernatant filtration, would reduce discrepancies between research studies and create a stronger evidence base for policymaking. This is urgent due to the increasing global mussel consumption, which is both an important source of nutrition and a sentinel species for marine pollution monitoring [65,105,106]. Aligning methods with food safety needs is essential to establish transparent, comparable microplastic exposure estimates from seafood.

## 5. Conclusions

This study discusses the compromise involved in selecting digestion methods for extracting microplastics from mussel tissues. KOH provides effective tissue digestion but has inconsistent microplastic recovery, particularly for PA fibres, and its temperature sensitivity may lead to underestimating MP contamination. In contrast, H_2_O_2_ demonstrated more stable recovery across different temperatures, polymer types and shapes, detecting both fragments and fibres.

This study demonstrates that digestion temperature and sample volume are as important as reagent choice for MP recovery. Optimal results are achieved at 50–60 °C with H_2_O_2_ (with 88.75 and 91.86%, respectively) and with 50 mL filtration. Temperatures above this may cause particle loss, and larger filtration volumes are not necessary. These findings indicate that focusing only on maximising digestion isn’t enough; improving particle recovery is also crucial with the flotation phase.

Developing a standardised digestion protocol tailored to food matrices and particle types is essential for improving microplastic exposure assessments. Future research should test both other seafood matrices and aged microplastics. Finally, setting standards will strengthen risk evaluation associated with MP-contaminated mussels and food safety.

## Figures and Tables

**Table 1:** Mean number and standard deviation of MPs recovered for each treatment applied.

Temperature	10% KOH	30% H_2_O_2_
Room Temperature	40 ± 0.58	68 ± 6.56
30 °C	42 ± 2.65	72 ± 2.08
40 °C	40	68 ± 6.66
50 °C	78 ± 0.58	70 ± 1.53
60 °C	58 ± 1.73	73 ± 7.09
70 °C	38 ± 4.58	73 ± 7.51

**Table 2:** Mean number and SD of fragments and fibres recovered at every level of filtration under each treatment applied.

		10% KOH		30% H_2_O_2_	
Temperature	Filtration Level	Fragments Recovered	Fibres Recovered	Fragments Recovered	Fibres Recovered
Room Temperature	50 mL	40	0	36 ± 2.12	32 ± 7.07
	100 mL	0	0	0	0
	150 mL	0	0	0	0
30 °C	50 mL	39 ± 2.12	3 ± 1.42	40	32 ± 2.83
	100 mL	0	0	0	0
	150 mL	0	1 ± 0.71	0	0
40 °C	50 mL	40	0	39 ± 1.42	27 ± 7.07
	100 mL	0	0	0	0
	150 mL	0	0	0	2 ± 3.54
50 °C	50 mL	38	38 ± 2.12	40	28
	100 mL	1 ± 1.41	2 ± 2.83	0	1 ± 0.71
	150 mL	0	1 ± 1.42	0	2 ± 1.41
60 °C	50 mL	34 ± 1.41	18 ± 3.54	36 ± 5.66	29 ± 2.83
	100 mL	2 ± 2.12	1 ± 1.42	4 ± 0.71	4 ± 4.95
	150 mL	0	5 ± 3.54	0	1 ± 2.12
70 °C	50 mL	30 ± 7.07	2 ± 1.42	34 ± 8.49	36 ± 5.66
	100 mL	3 ± 3.54	1 ± 1.42	0	0
	150 mL	0	2 ± 1.42	2 ± 2.12	1 ± 1.41

**Table 3:** Means and SD of MP dimensions at each level of filtration for each treatment applied.

		Minimum MP Dimensions (mm)			Maximum MP Dimensions (mm)		
	°C	50 mL	100 mL	150 mL	50 mL	100 mL	150 mL
**10% KOH**	RT	0.52 ± 0.98	-	-	2.75 ± 0.03	-	-
	30 °C	0.74 ± 0.53	-	1.54 ± 2.06	4.95 ± 1.49	-	2.36 ± 2.06
	40 °C	0.79 ± 0.09	-	-	5 ± 0.72	-	-
	50 °C	0.96 ± 0.42	0.24 ± 0.72	1.34 ± 0.13	4.62 ± 1.25	2.99 ± 0.63	1.85 ± 0.36
	60 °C	0.75 ± 0.06	0.89 ± 1.2	0.79 ± 0.86	4.08 ± 1.01	3.92 ± 1.98	3.84 ± 0.83
	70 °C	0.53 ± 0.58	0.72 ± 1.31	0.69 ± 0.88	2.93 ± 0.37	3.69 ± 1.32	3.65 ± 1.7
**30% H_2_O_2_**	RT	0.91 ± 0.46	-	-	4.72 ± 0.57	-	-
	30 °C	0.87 ± 0.15	-	-	5 ± 1.84	-	-
	40 °C	1.27 ± 0.2	-	2.75 ± 1.78	3.44 ± 0.4	-	5 ± 1.78
	50 °C	0.8 ± 0.39	1.01 ± 0.53	0.87 ± 0.17	4.36 ± 0.8	1.01 ± 0.71	1.88 ± 0.47
	60 °C	0.67 ± 0.11	0.53 ± 0.97	1.15 ± 0.18	4.34 ± 1.43	1.92 ± 0.06	2.18 ± 0.55
	70 °C	0.68 ± 0.39	-	0.59 ± 0.15	3.45 ± 0.5	-	3.21 ± 1.43

## Data Availability

The original contributions presented in the study are included in the article; further inquiries can be directed to the corresponding author.
